# Dr. Cordelia A. Greene

**Published:** 1905-03

**Authors:** 


Dr. Cordelia A. Greene, of Castile, N. Y., died at the Presbyterian Hospital, New York City, January 31, 1905, after a surgical operation, aged 74 years. Dr. Greene graduated from the medical department of Western Reserve University, Cleveland, in 1857. She succeeded her father in the management of a sanitarium at Castile, N. Y., which she successfully conducted for over forty years.

